# Association between Mental Health and Hand Hygiene Practices in Adults with Hypertension and Diabetes during the COVID-19 Pandemic: The 2020 Korea Community Health Survey

**DOI:** 10.3390/healthcare10101912

**Published:** 2022-09-29

**Authors:** Pius Kim, Hae Ran Kim

**Affiliations:** 1 Department of Neurosurgery, College of Medicine, Chosun University, Gwangju 61452, Korea; 2 Department of Nursing, College of Medicine, Chosun University, Gwangju 61452, Korea

**Keywords:** hypertension, diabetes, COVID-19, mental health, hand hygiene, health survey

## Abstract

The preventive measures against the spread of COVID-19 have negatively impacted the mental health of people with chronic diseases. This cross-sectional study investigated the association between mental health and hand hygiene practices in patients with hypertension (HTN) and diabetes mellitus (DM) (n = 74,296) during the COVID-19 pandemic. Their anxiety about contracting COVID-19 and death, depression, and hand hygiene practices were compared to that of controls. Multiple logistic regression analysis showed that the patients had higher anxiety and depression and poorer hand hygiene practices than controls. Anxiety about contracting COVID-19 was associated with increased handwashing before eating (aOR = 1.59), after using the restroom (aOR = 1.61), after returning from outdoors (aOR = 1.69), for at least 30 s (aOR = 1.45), and with soap or hand sanitizer (aOR = 1.43). However, depression was associated with decreased handwashing before eating (aOR = 0.50), after using the restroom (aOR = 0.51), after returning from outdoors (aOR = 0.51), for at least 30 s (aOR = 0.73), and with soap or hand sanitizer (aOR = 0.63). Anxiety about death showed similar results. Psychological support for people with chronic diseases in crisis situations may promote self-care activities such as hand hygiene for infection control.

## 1. Introduction

Severe acute respiratory syndrome coronavirus 2 (SARS-CoV-2) is responsible for the coronavirus disease (COVID-19) pandemic, which continues to cause considerable morbidity and mortality. Despite the widespread use of vaccines, the incidences, hospitalizations, and deaths due to COVID-19 remain public health concerns worldwide.

The prevalence of hypertension (HTN) and diabetes mellitus (DM) in Korea is steadily increasing. According to the results of the Korea National Health and Nutrition Examination Survey 2020, the prevalence of HTN and DM has increased to 22.9% and 10.7%, respectively, compared to those in 2019 [1]. Although the overall mortality rate of COVID-19 is low, it may adversely affect the prognosis of people with HTN and DM, which are both associated with disease severity [2]. According to recent meta-analysis studies, 18.6% of patients with COVID-19 have HTN and 11.9% have DM [3]. The risk of developing severe COVID-19 or death was found to be more than twice in people with HTN and DM compared to that in people without these diseases [4]. Since people with these conditions have weak immunity, they may have a higher risk of developing complications from COVID-19 compared to those without these diseases [5].

A pandemic can reduce the disease stability of people with chronic conditions, re-mind them about the risk of death, and negatively affect their mental health. Continuous exposure to excessive information about infectious diseases and measures to prevent the spread of the diseases such as social distancing, recommendations to stay at home, restrictions on visiting hospitals, and use of medicines may cause anxiety and depression in individuals with chronic diseases [6,7]. According to a previous study, 43.3% of people with HTN reported anxiety and 18.3% reported depression [8]. Similarly, 44.2% of patients with DM reported symptoms of anxiety and depression [9]. 

In a pandemic situation, when medical access is restricted, appropriate self-management is important for improving the survival and health of people with HTN and DM [10]. Maintaining good personal hygiene to prevent infection in patients with HTN and DM, who are immunocompromised, is an important part of self-management [11]. One of the essential recommendations issued by the World Health Organization (WHO) for public health during the COVID-19 pandemic was to wash hands frequently and properly [12]. General hand hygiene, such as frequent handwashing and use of soaps or sanitizers, may reduce an individual’s risk of contracting COVID-19 [13,14]. Therefore, maintaining good hand hygiene is one of the most important elements of self-management for the prevention of infectious diseases in patients with HTN and DM.

Anxiety and depression among patients with HTN and DM may affect hand hygiene. During the pandemic, high levels of anxiety were associated with high levels of personal hygiene, and depression was associated with low infection prevention measures [15,16]. A Chinese study found that anxiety was not associated with infection prevention measures, while depression was associated with poor hand hygiene [17]. Therefore, it is necessary to investigate the association of anxiety and depression with hand hygiene in Korean patients with HTN and DM during the COVID-19 pandemic. This information may help establish national public health policies and develop effective strategies to combat the health threats to patients with HTN and DM.

Inadequate self-management practices including poor personal hygiene and poor mental health status have been reported during the pandemic among patients with HTN and DM [18,19]. However, data on mental health and hand hygiene practices among patients with HTN and DM living in Korean communities are limited. The purpose of this study was to compare the mental health problems and hand hygiene practices among patients with HTN and DM living in Korean communities with that of control group without these diseases, and to investigate the relationship between mental health problems and hand hygiene practices in patients with HTN and DM.

## 2. Materials and Methods

### 2.1. Data Source and Study Population

The 2020 Korea Community Health Survey (KCHS) was conducted by the Korea Centers for Disease Prevention and Control. The KCHS has been used to assess the level of regional health by collecting health statistics from cities, counties, and districts, as well as to establish a regional healthcare plan. Survey items and output indices were validated by the operating committee after getting feedback from policy departments and local governments [20]. Stratified cluster sampling and systematic sampling methods were used to select the sample areas and households, respectively. Target participants were adults aged 19 years or older. Trained health interviewers visited each household to conduct the survey, and a 1:1 face-to-face computer-assisted personal interview was conducted [20]. All interviewers were trained in the prevention of COVID-19. Only the interviewers who tested negative for COVID-19 participated in the survey, and their health conditions such as COVID-19-related symptoms were checked every day. During the survey, the interviewers disinfected their hands, wore a mask, and maintained two arms’ length distance from the participants. Participants were asked to check their body temperature and health condition and wear a mask before the survey. If a sample household had a patient with COVID-19 or a self-quarantined individual, the sample was replaced with another. 

The Institutional Review Board of the Korea Centers for Disease Control and Prevention approved the survey protocol (2016-10-01-P-A). Detailed information is provided on the KCHS website (https://chs.kdca.go.kr/chs/ accessed on 20 August 2022). Individuals with HTN or DM were defined as those who answered “yes” to the following question: “Have you ever been diagnosed with hypertension or diabetes by a physician?” An exact sex-matched comparison group was selected among the participants without a history of HTN and DM. Sex is a known potential confounding factor for the outcome of interest [21]. Considering 900 people living in each of the 255 health centers, a total of 229,269 adults participated in the 2020 KCHS, and 74,296 (32.4%) participants were identified as having HTN or DM. 

### 2.2. Mental Health 

Mental health analysis included anxiety about contracting COVID-19 and death due to the same, and depression. Anxiety was measured with the following questions: “I am anxious about getting infected with COVID-19” (anxiety about contracting COVID-19) and “I am anxious that I might die if I get infected with COVID-19” (anxiety about death due to COVID-19). The Patient Health Questionnaire-9 was used to assess depression. The questionnaire contains nine items, and participants can respond to each item on a 4-point Likert-type scale ranging from 0 to 3. Scores can range from 0 to 27, with scores greater than 10 classified as depression [22]. 

### 2.3. Hand Hygiene Practice

Frequent handwashing before eating, after using the restroom, and after returning from the outdoors were measured with the following question: “How frequently did you wash your hands during the last week?” Response options included “always,” “often,” “sometimes,” and “rarely.“ “Always” and “often” were classified as yes, while other responses were classified as no. The frequency of handwashing for more than 30 s was defined as yes when the answers were “always” or “often” to the question: “When washing your hands, did you thoroughly wash under running water for at least 30 s?” (possible answers were: always, often, sometimes, or rarely). Frequent handwashing with soap or hand sanitizer was measured with the following question: “How frequently did you use soap or hand sanitizer when you washed your hands?” Response options included “always,” “often,” “sometimes,” “rarely,“ and “never.” “Always” and “often” were classified as yes, while other responses were classified as no.

### 2.4. Covariate

The general characteristics included sex, age (19–29, 30–49, 50–64, ≥65 years), educational level (≤middle school, high school, ≥college), family type (living with spouse, living alone, others), monthly income (high, middle–high, middle–low, low), location of residence (urban, rural), subjective health status (good, poor), current smoker (no, yes), current alcohol user (no, yes), duration of disease (≤5 years, >5 years), and disease treatment (non-pharmacologic methods [exercise, diet], pharmacologic therapy). For disease treatment, responses including both options simultaneously were possible. Pharmacologic therapy included hypertension medication, insulin, and oral hypoglycemic agents.

### 2.5. Statistical Analyses

SAS version 9.2 (SAS Institute, Cary, NC, USA) was used to analyze the data. The analysis accounted for the complex and multistage sampling design of the KCHS. Comparisons of numbers and proportions reflecting mental health and hand hygiene practices between participants with HTN and DM and the sex-matched control group were performed using chi-square test. Multiple logistic regression analysis was used to assess whether mental health and hand hygiene practices significantly differed between the two groups after adjusting for the covariates. Associations between mental health and hand hygiene practices in people with HTN and DM were investigated using multiple logistic regression analyses. Statistical significance was set at *p* < 0.05.

## 3. Results

Approximately 90% of the participants were over the age of 50 years. Additionally, 12.2% of the participants with HTN and DM reported a duration of the disease greater than 5 years. Among the participants, 31.4% reported using non-pharmacologic methods for disease management, and 95.5% were undergoing pharmacologic therapy (Table 1). 

Anxiety about contracting COVID-19 (73.9% vs. 69.0%, adjusted odds ratio [aOR] = 1.04, 95% confidence interval [CI] = 1.02–1.07), anxiety about death due to COVID-19 (54.3% vs. 40.7%, aOR = 1.11, 95% CI = 1.09–1.14), and depression (3.5% vs. 2.6%, aOR = 1.11, 95% CI = 1.03–1.20) were significantly higher in those with HTN and DM than in those without the diseases (Table 2). 

The proportion of participants washing their hands before eating, after using the restroom, and after returning from the outdoors, for at least 30 s, and with soap or hand sanitizer during the COVID-19 pandemic was significantly lower (*p* < 0.001) in those with HTN and DM than in those without the diseases (Table 3).

The results of multiple logistic regression analyses revealed that participants with anxiety about contracting COVID-19 were more likely to wash their hands before eating (aOR = 1.59, 95% CI = 1.49–1.69), after using the restroom (aOR = 1.61, 95% CI = 1.51–1.72), and after returning from outdoors (aOR = 1.69, 95% CI = 1.58–1.80), and for at least 30 s (aOR = 1.45, 95% CI = 1.39–1.51) with soap or hand sanitizer (aOR = 1.43, 95% CI = 1.36–1.50) compared to those without anxiety. Participants with anxiety about death due to COVID-19 were more likely to wash their hands before eating (aOR = 1.45, 95% CI = 1.36–1.55), after using the restroom (aOR = 1.39, 95% CI = 1.30–1.48), and after returning from outdoors (aOR = 1.57, 95% CI = 1.48–1.68), and for at least 30 s (aOR = 1.45, 95% CI = 1.39–1.50) with soap or hand sanitizer (aOR = 1.31, 95% CI = 1.25–1.36) compared to those without anxiety. Participants with depression were less likely to wash their hands frequently before eating (aOR = 0.50, 95% CI = 0.44–0.57), after using the restroom (aOR = 0.51, 95% CI = 0.45–0.59), and after returning from outdoors (aOR = 0.51, 95% CI = 0.45–0.58), and for at least 30 s (aOR = 0.73, 95% CI = 0.67–0.80) with soap or hand sanitizer (aOR = 0.63, 95% CI = 0.57–0.70) compared to those without depression (Table 4).

## 4. Discussion

In this study, the association between mental health and hand hygiene practices of patients with HTN and DM during the COVID-19 pandemic were compared to that of controls. According to the results of this study, Korean adults with HTN and DM had a higher risk of developing anxiety about contracting COVID-19 and death due to COVID-19, depression, and a lower tendency of following hand hygiene practices than the control group without these diseases. Among the patients with HTN and DM, anxiety about the infection and death was associated with an increase in good hand hygiene practices, and depression was associated with a decrease in good hand hygiene practices.

Preventive measures to reduce the spread of COVID-19 may lead to anxiety and de-pression in people with chronic conditions. In studies conducted during the COVID-19 pandemic, HTN and DM were associated with increased anxiety due to COVID-19 [6,8,9]. Similarly, in this study, anxiety about contracting COVID-19 and death due to it, and depression were higher in people with HTN and DM compared to the controls without HTN and DM. It is known that people with chronic diseases are more vulnerable to psychological stress when faced with an unpredictable disease such as COVID-19 [6]. Isolation due to social distancing during the COVID-19 pandemic can negatively impact maintenance of a healthy lifestyle, the prescribing and taking of medications, and accessing health care for patients with chronic diseases [23], which may lead to mental health problems [7]. Providing accurate health information, psychological first aid, and counseling to patients with HTN and DM under social isolation during the pandemic may help improve their mental health [24,25]. 

Similar to the results of the 2017 community health survey before the spread of COVID-19 [11], the prevalence of good hand hygiene practices was significantly lower in those with HTN and DM compared to the sex-matched control group. Approximately 20% of patients with HTN and DM reported not washing their hands for more than 30 s and 14% reported not washing their hands with soap. SARS-CoV-2 caused severe cardiovascular damage during the pandemic [5], and HTN and DM have been reported to be associated with the fatal outcome of COVID-19 across all ages [4]. Handwashing 6–10 times per day is linked to a reduced risk of contracting COVID-19 [13]. The Centers for Disease Control and Prevention (CDC) recommends handwashing with soap for at least 20 s to prevent the spread of COVID-19 [26]. As SARS-CoV-2 can survive outside the human body for a long time, particular emphasis is placed on handwashing with soap to reduce exposure to the virus when a person sneezes and coughs in public places, and while using public toilets [27]. Proper handwashing for patients with cardiovascular issues is the most basic health maintenance behavior to prevent contracting COVID-19. This result may be explained as follows. The majority of patients with HTN and DM are of old age, lower education level, and have lower monthly income. These low social and financial circumstances are known low self-management factors for maintaining health [28]. For example, in older adults and in those with lower education levels, reduced risk perception of COVID-19 due to low cognitive function may lead to reduced health self-management [29,30]. Therefore, a health management program is needed for patients with chronic diseases who are socially marginalized or have financial crises. In addition, patients with HTN and DM need to be educated on the importance of good hand hygiene practices as they are more susceptible to getting infected with COVID-19 [31]. Further research is needed as the 2020 KCHS database did not have information on where to wash hands and on handwashing procedures. 

Among people with HTN and DM, those who reported anxiety about contracting COVID-19 and death due to the same had a significantly higher rate of handwashing before eating, after using the restroom, and after returning from outdoors, for at least 30 s, and with soap or hand sanitizer than those who did not report anxiety. In contrast, a study conducted in China reported that state and trait anxieties were not associated with preventive behavior [17]; a Japanese study demonstrated that participants with anxiety were less likely to engage in preventive behavior [32]. This may be explained by the differences in anxiety assessment tools. Anxiety assessment tools used in previous studies measured the degree of anxiety that could cause problems in daily life, and included the Generalized Anxiety Disorder 7-item scale and the State-Trait Anxiety Inventory. However, the tool used in the present study measured the perceived risk of COVID-19. Risk perception has been reported to increase fear and positively influence preventive behavior [33]. Anxiety about contracting COVID-9 and death due to it may raise awareness about disease prevention and reduce the disease spread [34]. For example, patients with chronic diseases, who are anxious about contracting the infection, are likely to choose handwashing as a safety mechanism to control infection [35]. Conversely, those who reported depression had significantly poorer hand hygiene practices than those who did not. Our findings are consistent with those of a previous study suggesting that depressive symptoms may inhibit preventive actions against the COVID-19 pandemic [17]. People with depression have difficulty in exhibiting adaptive behaviors for appropriate coping in crisis situations. During the pandemic, implementation of preventive actions may worsen in patients with HTN and DM who are vulnerable to psychological problems. The group with depression and those with chronic diseases who experience psychological distress participate less in proper handwashing than the group without depression. Additionally, the group with depression may choose poor hand hygiene practices during the COVID-19 pandemic. Therefore, more attention to manage depression in patients with HTN and DM can promote handwashing and prevent the spread of infectious diseases. The current findings show that community agencies should screen patients with chronic diseases for mental health problems as they are less likely to adhere to hand hygiene practices, and develop psychological support programs and campaigns targeting them.

This is the first large-scale study to report an association between mental health problems and hand hygiene practices in Korean adults with HTN and DM during the COVID-19 pandemic. Many previous studies have emphasized the need for active mental health management to enable infection prevention behavior in patients with chronic diseases [32,33,35]. Taken together, these results suggest that the negative mental health of patients with non-communicable diseases (NCD) due to the impact of COVID-19 adversely affects healthy behavior. Therefore, we need to pay more attention to the mental health of patients with NCD. Such identification may help health systems prioritize those who may be more negatively impacted during an epidemic.

The present study has several limitations. First, the KCHS did not collect detailed clinical data such as the severity of HTN and DM, hospitalization experience, current disease status, blood test results, and COVID-19-related medical history (infection, treatment, or isolation). Further studies are needed to investigate the mental health and hand hygiene practices associated with the clinical status of patients with HTN and DM. Second, a self-reported method was used to measure anxiety and depression during the COVID-19 pandemic. Social desirability bias may have influenced participants to underreport mental problems and overreport hand hygiene practices. Third, as the KCHS focused on the community population, patients in general hospitals or nursing homes were not included. Although handwashing is emphasized in both community and hospital settings, in a pandemic situation, community-based approaches can improve the self-management of people with chronic conditions [18]. Fourth, it was difficult to establish a causal relationship between mental health problems and hand hygiene practices during the COVID-19 pandemic because the KCHS data are cross-sectional. Longitudinal data are needed to assess the impact of changes in the social environment due to COVID-19. Fifth, our data included participants only within South Korea; therefore, it is difficult to generalize the results to other countries. However, mental health problem has been found to have stable associations with COVID-19 preventive behaviors in several studies [15,16,17]; therefore, the results of this study can be applied to improve hand hygiene practices in patients with various chronic diseases. 

## 5. Conclusions

This study suggests that mental health problems in Korean patients with HTN and DM may have a negative impact on their hand hygiene practices which are necessary to prevent getting infected with COVID-19. It is essential to develop community-based support programs that include the management of psychological problems in people with chronic diseases during the pandemic. For people with HTN and DM who are at high risk of contracting infectious diseases, these psychological support programs may promote self-management activities such as hand hygiene practices.

## Figures and Tables

**Table 1:** Characteristics of adults with HTN or DM and controls.

Characteristics	With HTN or DM (*N* = 74,296)		Without HTN and DM (*N* = 74,296)	
	N	%	N	%
Sex				
Male	34,030	45.8	34,030	45.8
Female	40,266	54.2	40,266	54.2
Age				
19–29	427	0.6	12,431	16.7
30–49	6119	8.2	26,456	35.6
50–64	23,593	31.8	21,700	29.2
≥65	44,151	59.4	13,705	18.5
Education level				
≤Middle school	43,761	59.0	16,799	22.6
High school	19,308	26.0	28,229	38.0
≥College	11,120	15.0	29,185	39.4
Family type				
Living with spouse	31,225	42.0	18,160	24.4
Living alone	16,151	21.7	9495	12.8
Others	26,918	36.3	46,637	62.8
Monthly income				
High	18,410	24.8	27,827	37.5
Middle–high	9399	12.7	16,838	22.7
Middle–low	21,911	29.5	20,077	27.0
Low	24,576	33.0	9554	12.8
Location of residence				
Urban (dong)	34,518	46.5	45,293	61.0
Rural (eup or myeon)	39,778	53.5	29,003	39.0
Subjective health status				
Good	23,242	31.3	41,616	56.0
Poor	51,051	68.7	32,677	44.0
Current smoker				
No	63,964	86.1	60,104	80.9
Yes	10,318	13.9	14,181	19.1
Current alcohol user				
No	48,736	65.6	37,050	49.9
Yes	25,554	34.4	37,245	50.1
Duration of disease				
≤5 years	65,197	87.8		
>5 years	9099	12.2		
Disease treatment				
Non-pharmacologic methods (exercise, diet)	23,317	31.4		
Pharmacologic therapy	70,964	95.5		

Data are expressed as the number (%); all missing values are not included; HTN, hypertension; DM, diabetes mellitus.

**Table 2:** Mental health status during the COVID-19 pandemic among people with HTN and DM compared to controls.

Characteristics	With HTN or DM		Without HTN or DM		*p* *
	N	%	N	%	
Mental Health Status					
Anxiety about COVID-19 infection					
No	19,366	26.1	23,014	31.0	<0.001
Yes	54,896	73.9	51,264	69.0	
aOR (95% CI) for anxiety about COVID-19 infection ^a^	1.04 (1.02–1.07)		1.00		
Anxiety about death due to COVID-19 infection					
No	33,941	45.7	44,013	59.3	<0.001
Yes	40,274	54.3	30,237	40.7	
aOR (95% CI) for anxiety about death ^a^	1.11 (1.09–1.14)		1.00		
Depression (PHQ-9)					
<10	71,345	96.5	72,151	97.4	<0.001
≥10	2578	3.5	1918	2.6	
aOR (95% CI) for ≥10 ^b^	1.11 (1.03–1.20)		1.00		

All missing values are not included; COVID-19 = coronavirus disease 2019; HTN, hypertension; DM, diabetes mellitus; aOR, adjusted odds ratio; CI, confidence interval; * a chi-squared test; ^a^ adjusted for sex, age, educational level, family type, monthly income, location of residence, subjective health status, current smoker, and current alcohol user; ^b^ additionally adjusted for anxiety about contracting COVID-19 and death due to COVID-19.

**Table 3:** Hand hygiene practices during the COVID-19 pandemic among people with HTN and DM compared to controls.

Characteristics	With HTN or DM		Without HTN or DM		*p* *
	N	%	N	%	
Handwashing before eating					
No	4507	6.1	3696	5.0	<0.001
Yes	69,787	93.9	70,598	95.0	
aOR (95% CI) for handwashing before eating ^a^	1.00 (0.95–1.06)		1.00		
Handwashing after using the restroom					
No	4298	5.8	2484	3.3	<0.001
Yes	69,993	94.2	71809	96.7	
aOR (95% CI) for handwashing after using the restroom ^a^	1.09 (1.03–1.16)		1.00		
Handwashing after returning from the outdoors					
No	4381	5.9	2655	3.6	<0.001
Yes	69,857	94.1	71,613	96.4	
aOR (95% CI) for handwashing after returning from the outdoor ^a^	0.93 (0.88–0.99)		1.00		
Handwashing for at least 30 s ^a^					
No	14,803	19.9	12,252	16.5	<0.001
Yes	59,487	80.1	62,029	83.5	
aOR (95% CI) for handwashing for at least 30 s ^a^	1.03 (0.99–1.06)		1.00		
Handwashing with soap or hand sanitizer					
No	10,334	14.0	6180	8.3	<0.001
Yes	63,745	86.0	67,978	91.7	
aOR (95% CI) for handwashing with soap or hand sanitizer ^a^	1.03 (0.99–1.07)		1.00		

COVID-19 = coronavirus disease 2019; HTN, hypertension, DM, diabetes mellitus; aOR, adjusted odds ratio; CI, confidence interval; * a chi-squared test; ^a^ adjusted for sex, age, educational level, family type, monthly income, location of residence, subjective health status, current smoker, and current alcohol user.

**Table 4:** Association between psychological problems and hand hygiene practices among people with HTN or DM during the COVID-19 pandemic.

Characteristics	Anxiety About COVID-19 Infection ^a^	Anxiety About Death Due to COVID-19 Infection ^a^	Depression ^b^
	aOR (95% CI)	aOR (95% CI)	aOR (95% CI)
Handwashing before eating	1.59 (1.49–1.69)	1.45 (1.36–1.55)	0.50 (0.44–0.57)
Handwashing after using the restroom	1.61 (1.51–1.72)	1.39 (1.30–1.48)	0.51 (0.45–0.59)
Handwashing after returning from outdoors	1.69 (1.58–1.80)	1.57 (1.48–1.68)	0.51 (0.45–0.58)
Handwashing for at least 30 s	1.45 (1.39–1.51)	1.45 (1.39–1.50)	0.73 (0.67–0.80)
Handwashing with soap or hand sanitizer	1.43 (1.36–1.50)	1.31 (1.25–1.36)	0.63(0.57–0.70)

All missing values are not included; COVID-19 = coronavirus disease 2019; HTN, hypertension; DM, diabetes mellitus; aOR, adjusted odds ratio; CI, confidence interval; ^a^ adjusted for sex, age, educational level, family type, monthly income, location of residence, subjective health status, current smoker, and current alcohol user; ^b^ additionally adjusted for anxiety about contracting COVID-19 and death due to COVID-19.

## Data Availability

All data are available on request from the corresponding author.
